# Association between Sarcopenia and Insulin-Like Growth Factor-1, Myostatin, and Insulin Resistance in Elderly Patients Undergoing Hemodialysis

**DOI:** 10.1155/2022/1327332

**Published:** 2022-03-23

**Authors:** Novira Widajanti, SoebagijoAdi Soelistijo, Usman Hadi, Mochammad Thaha, Aditiawardana, Widodo, Hadiq Firdausi, YafanitaIzzati Nurina, MiraDelima Asikin, Hersih Srinowati, NoerHalimatus Syakdiyah

**Affiliations:** ^1^Department of Internal Medicine, Dr. Soetomo Hospital-Faculty of Medicine Airlangga University, Surabaya 60286, Indonesia; ^2^Geriatric Division-Department of Internal Medicine, Dr. Soetomo Hospital-Faculty of Medicine Airlangga University, Surabaya 60286, Indonesia; ^3^Endocrinology, Metabolism and Diabetes Division, Department of Internal Medicine, Dr. Soetomo Hospital-Faculty of Medicine Airlangga University, Surabaya 60286, Indonesia; ^4^Nephrology Division, Department of Internal Medicine, Dr. Soetomo Hospital-Faculty of Medicine Airlangga University, Surabaya 60286, Indonesia; ^5^Faculty of Medicine, Universitas Airlangga, Surabaya 60132, Indonesia

## Abstract

Sarcopenia is common in hemodialysis patients, especially in the elderly patients undergoing hemodialysis. Various factors may contribute to the occurrence of sarcopenia, such as anabolic and catabolic imbalance. This study aims to investigate the correlation of insulin-like growth factor-1 (IGF-1) levels as an anabolic factor, myostatin levels, and insulin resistance as catabolic factors with sarcopenia in the pathogenesis of sarcopenia in elderly patients undergoing hemodialysis. A total of 40 subjects aged 60 years or more who undergoing hemodialysis in Dr. Soetomo Hospital Surabaya were included in this cross-sectional study. Sarcopenia was diagnosed according to Asian Working Group Sarcopenia 2019 criteria. IGF-1, myostatin, and insulin resistance levels were measured once before hemodialysis. Subjects with sarcopenia diagnosis were 33 (82.5%), that is, 19 (47.5%) men and 14 (35%) women. There were 28 (70%) of the subjects diagnosed with severe sarcopenia. Furthermore, there were significant differences in the characteristics and geriatric parameters between the sarcopenia and nonsarcopenia groups. There were differences between the two groups in hemoglobin levels, IGF-1 levels, myostatin levels, homeostasis model assessment-insulin resistance (HOMA-IR) levels, muscle mass, handgrip strength, body mass index status, mini nutritional assessment status, and physical activity scale for elderly status (all *p* < 0.05). Correlation analyses showed that IGF-1 levels negatively correlated with sarcopenia status in elderly patients undergoing hemodialysis (*p* < 0.05). On the contrary, myostatin and HOMA-IR levels were positively correlated with sarcopenia status in elderly patients undergoing hemodialysis (all *p* < 0.05). Based on this recent study, IGF-1, myostatin, and insulin resistance were significantly correlated with sarcopenia in elderly patients undergoing hemodialysis.

## 1. Introduction

Sarcopenia is a chronic condition characterized by losing muscle mass, strength, and function due to aging. It is a common complication in adults with CKD, especially among patients with end-stage renal disease undergoing hemodialysis (HD) [1]. In addition, sarcopenia in CKD patients leads to a sedentary lifestyle and a lower quality of life, as well as an increased risk of cardiovascular problems, morbidity, and mortality [2].

Primary sarcopenia is defined when aging is the primary factor of muscle loss and function. Meanwhile, secondary sarcopenia is determined when one or more factors are present such as sedentary behavior, malnutrition, deconditioning, organ failure, and inflammatory diseases [3]. In elderly sarcopenia, changes in circulating anabolic (insulin, insulin-like growth factors, growth hormone) and catabolic (TNF-Alfa, cortisol, catecholamine, glucagon, cytokines) molecules affect the ratios of anabolism and catabolism. Both muscle and general metabolism undergo significant alterations as a result of this [4].

IGF-1 is an anabolic hormone essential for skeletal muscle development, growth, differentiation, and maintenance [5]. Otherwise, myostatin is a catabolic factor that belongs to the transforming growth factor (TGF) superfamily and acts as a negative regulator of muscle growth [6]. In chronic conditions, myostatin levels are negatively correlated with muscle atrophy [7]. Insulin resistance and reduced insulin levels are catabolic conditions that cause muscle protein synthesis decrease and concurrent muscle protein breakdown. In addition, insulin resistance can exacerbate catabolic diseases like end-stage chronic kidney disease [8].

In this study, we investigated the correlation between IGF-1 levels, myostatin levels, and insulin resistance and sarcopenia in elderly patients undergoing HD. The findings could suggest comprehensive treatment and a health-management strategy to combat the problems of disease.

## 2. Materials and Methods

### 2.1. Study Design and Subjects

A cross-sectional study was conducted on December 2020-January 2021 at Hemodialysis Center Unit, Dr. Soetomo General Academic Hospital, Surabaya, East Java, Indonesia. A minimum sample of 38 patients was determined by estimating the sample size for the difference between the two correlation formulae [9]:

(1) $$\begin{array}{c} N={\left[\frac{\left(Z_{\alpha }+Z_{\beta }\right)}{C}\right]}^{2}+3, \end{array}$$

*Z*_*α*_ = the standard normal deviate for *α*, with the alternative hypothesis being two-sided, *α* value 0.05 was set, then *Z*_*α*_ = 1.96, *Z*_*β*_ = the standard normal deviate for *β*, *β* value 0.10 was set then *Z*_*β*_ = 1.282, *C* = 0.5 × ln [(*1* + *r*)/(*1* − *r*)], *r* = expected correlation coefficient, *r* value was 0.5, *N* = Total number of subjects required.

Sampling was carried out by total sampling with inclusion criteria as follows: (i) elderly (≥60 years) undergoing HD on December 2020-January 2021 at least twice a week in the last three months; (ii) able to communicate. Patients with (i) acute infection disease; (ii) severe cognitive impairment (Mini-Mental State Examination (MMSE) score <18); (iii) activities of daily living (ADL) score <8; (iv) history of recent fractures or history of surgery due to fracture less than six months; (v) history of severe motor disorders (paresis, paralysis); (vi) Diabetes mellitus on treatment; (vii) cirrhosis hepatic; (viii) history of cancer; (ix) corticosteroid, oral contraception, antipsychotic, and antiviral (protease inhibitor) therapy, were excluded.

The effect of all human studies has been reviewed by a relevant institutional ethics committee (0112/KEPK/XII/2020) before the study was conducted and has been performed following the ethical standards of the Declaration of Helsinki. All respondents gave their written informed consent before their inclusion in the study. Information for informed consent was provided before the informed consent form was signed. Details that might disclose the identity of the respondents were omitted.

### 2.2. Geriatric Profiles Assessment Test

The geriatric biopsychosocial profiles of the subjects were obtained using questionnaires. (a) Nutritional risk status was assessed using Mini Nutritional Assessment (MNA) full form questionnaire: normal (scores >23.5), at risk of malnutrition (scores 17–23.5), malnourished (scores <17) [10]. MMSE questionnaire to assess five different areas in cognitive functions such as orientation, registration, attention and calculation, recall, and language: normal (scores 24–30), mild cognitive impairment (scores 18–23), severe cognitive impairment (scores<18) [11]. Short form of Geriatric Depression Scale (GDS) questionnaire to assess depression category with 15 item questions: normal (scores <5), probable depression (scores 5–9), and depression (scores ≥10) [12]. Barthel Activity of Daily Living (ADL) scale to evaluate independent living: totally dependent (scores 0–4), heavily dependent (scores 5–8), moderately dependent (scores 9–11), mildly dependent (scores 12–19), independent (scores 20) [13]. Physical Activity Scale for Elderly (PASE) questionnaire to assess physical activity status over the past week with 12 item questions [14]. The PASE score was stratified in tertiles: 0 to 40 (sedentary), 41 to 90 (light physical activity) and more than 90 (moderate to intense activity).

### 2.3. Sarcopenia Diagnosis

Sarcopenia was diagnosed based on AWGS 2019 criteria. Sarcopenia was defined as low muscle mass with either low muscle strength or low physical performance. Severe sarcopenia was defined as low muscle mass with low muscle strength and low physical performance [15].

Muscle mass was evaluated using the Body Composition Monitor Omron Karada scan model HBF-362 (Omron Corporation, Kyoto, Japan). Participants wore light clothes and stood barefoot on the instrument. Body mass, appendicular skeletal muscle mass (ASM), and height were measured accurately to 0.1 kg and 0.1 cm, respectively. Muscle mass was obtained from calculations of ASM, which represent the mass of the four limbs (kg) divided by the square of the height in meters (m^2^) [15]. The AWGS 2019 cut-offs for low muscle mass in sarcopenia diagnosis are <7.0 kg/m^2^ in men and <5.7 kg/m^2^ in women [15], whereas body mass index (BMI) was calculated as weight in kilograms divided by the square of the height in meters (kg/m^2^). It was categorized into four groups according to the Asian-Pacific cut-off points, underweight (<18.5 kg/m^2^), normal weight (18.5–22.9 kg/m^2^), overweight (23–24.9 kg/m^2^), and obese (≥25 kg/m^2^) [16].

Muscle strength was defined as the maximum strength of muscle to hold a load. Muscle strength was measured by handgrip strength (HGS). Measurement was performed on the maximum grip strength of the dominant arm. A handgrip dynamometer from Takei TKK 5001 Grip-A was used for measurement and expressed on a number scale indicated by the peak hold needle in kilograms (kg). AWGS cut-off for low muscle strength is handgrip strength value < 28 kg for men and <18 kg for women [15].

Physical performance was defined as a form of mobility that is a part of physical function. In this study, physical performance was evaluated by measuring the gait speed from 6 meter walking test. Participants were instructed to walk at their usual pace to 6 meters distance and measured speed. The AWGS 2019 cut-off for low physical performance is < 1.0 m/sec [15].

### 2.4. Laboratory and Hormonal Parameters

Laboratory parameters were taken from the patients' medical records. Homeostasis Model Assessment-Insulin Resistance (HOMA-IR) was calculated from fasting plasma insulin levels (mU/L) × fasting blood glucose levels (mg/dL) divided by 405 [17]. Blood samples were taken after the patient fasted for 8 hours. Fasting plasma insulin was measured using Electrochemiluminescence immunoassay (ECLIA) method, while fasting blood glucose was measured using the Glucose Oxidase-Peroxidase Aminoantipyrine (GOD-PAP) enzymatic colorimetric method. Myostatin levels and IGF-1 levels were measured using the Rayto tool with Enzyme-linked Immunosorbent Assay (ELISA) method before HD.

### 2.5. Statistical Analysis

The collected data were processed with SPSS 23.0 for windows (IBM Corp., Armonk, NY, USA). Descriptive data were presented in the form of text and tables as appropriate. Report means (standard deviation) for normally distributed data and medians (interquartile ranges) for skewed data, whereas categorical variables were presented as a proportion (frequency and percentage). Shapiro-Wilks test was used to assess the data normality. The Kruskal-Wallis test was used to compare three groups, and the Mann-Whitney *U* test was used to compare two groups. The correlation between sarcopenia and independent variables was evaluated using the Spearman test. *p* value less than 0.05 was considered to be statistically significant.

## 3. Results

There were 40 eligible subjects in this study. Subjects with sarcopenia diagnosis were 33 (82.5%), that is, 19 (47.5%) men and 14 (35%) women. There were 28 (70%) of the subjects diagnosed with severe sarcopenia.

### 3.1. Characteristics and Factors Associated with Sarcopenia

Baseline characteristics of subjects are shown in Table 1, and the geriatric profiles are shown in Table 2. There were differences between the two groups in hemoglobin levels, IGF-1 levels, myostatin levels, HOMA-IR levels, muscle mass, and HGS (all *p* < 0.05) (see Table 1). With regard to geriatric profiles, there were differences between the two groups in BMI status, MNA status, and PASE status (all *p* < 0.05) (see Table 2).

**Figure 1:**
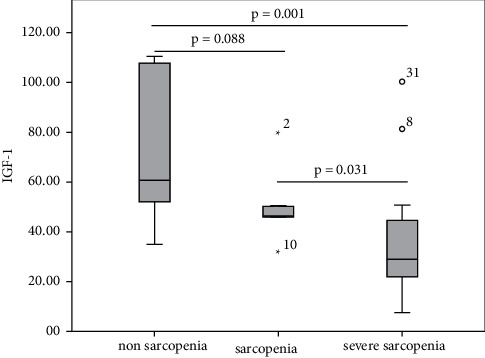
Comparisons of IGF-1 levels in sarcopenia status group.

**Figure 2:**
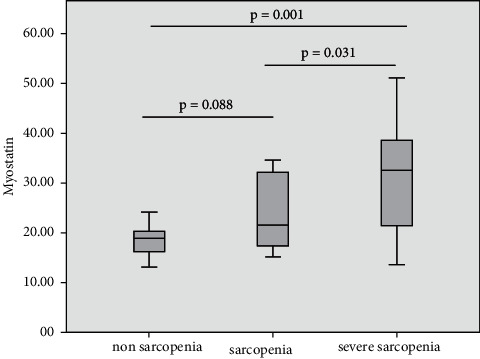
Comparisons of myostatin levels in sarcopenia status group.

**Figure 3:**
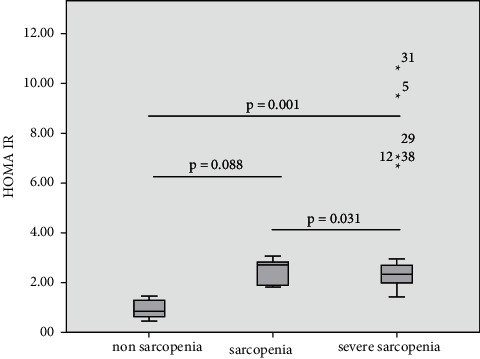
Comparisons of HOMA-IR levels in sarcopenia status group.

IGF-1, myostatin, and HOMA-IR levels were divided into the nonsarcopenia, sarcopenia, and severe sarcopenia groups. Comparisons of IGF-1, myostatin, and HOMA-IR in the sarcopenia status group are shown in Figures 1–3. IGF-1 levels in nonsarcopenia group were higher than sarcopenia and severe sarcopenia group (60.57 [51.31–65.33] vs 46.23 [38.85–65.33] vs 29.01 [21.77–44.96] ng/ml, respectively; *p* = 0.001) (see Figure 1). Myostatin levels and HOMA-IR in nonsarcopenia group were lower than sarcopenia and severe sarcopenia group (18.87 [13.48–20.87] vs 21.48 [16.1–33.33] vs 32.48 [21.38–38.90] ng/ml, *p* = 0.014) (see Figure 2) and 0.87 [0.56–1.33] vs 2.73 [1.88–2.96] vs 2.35 [1.93–2.72], respectively; *p* < 0.001(see Figure 3)).

### 3.2. Correlations of IGF-1, Myostatin, and HOMA-IR with Sarcopenia Status

Correlations of IGF-1, myostatin, and HOMA-IR with sarcopenia are shown in Table 3. The patients' IGF-1 levels were negatively correlated with sarcopenia status (*r* = -0.604, *p* < 0.001). Otherwise, the patients' myostatin levels (*r* = 0.462, *p* = 0.003) and HOMA-IR (*r* = 0.496, *p* = 0.001) were positively correlated with sarcopenia status.

## 4. Discussion

Sarcopenia in elderly patients undergoing HD is a serious health issue because it increases morbidity and mortality in patients with CKD. The purpose of the study was to understand IGF-1, myostatin, and insulin resistance levels correlated with sarcopenia and the role of IGF-1, myostatin, and insulin resistance in the occurrence of sarcopenia in elderly patients undergoing HD. The results suggested that IGF-1, myostatin, and insulin resistance were correlated with sarcopenia in elderly patients undergoing HD. In addition, patients in the sarcopenia group had lower IGF-1 levels but higher myostatin and HOMA-IR levels than the nonsarcopenia group.

The prevalence of sarcopenia in Asia is around 4.1%–16.3% in women and 5.5%–25.7% in men [15]. Although there is a lack of national data regarding the prevalence of sarcopenia in Indonesia, especially in CKD patients, a study in Surabaya, East Java, Indonesia, on 320 participants showed a 13.9% prevalence in older men and 27.9% in older women based on AWGS 2014 parameters [18]. In the present study, sarcopenia in elderly patients undergoing HD was 19 (47.5%) in men and 14 (35%) in women. A cohort study conducted by Mori et al. reported the prevalence of sarcopenia among HD patients in Shirasagi Hospital, Japan, which was determined to be 40% based on AWGS 2014 parameters [19], Meanwhile, a study in Brazil reported that the prevalence of sarcopenia in patients with CKD, and not yet HD, was 11.9% and 28.7% using the EWGSOP and FNIH criteria [20].

The etiology of sarcopenia is multifactorial although many factors are not modifiable. This has led to increasing interest in the influence of lifestyle, particularly the effects of modifiable factors, such as nutritional status [21]. This study suggested that there were hemoglobin levels, BMI status, and MNA status differences in the occurrence of sarcopenia, indicating that the nutritional status was associated with sarcopenia. A few studies have suggested that malnutrition increases the risk of sarcopenia due to reduced muscle protein synthesis [22]. Nutritional interventions could make an important contribution to preventing sarcopenia [23].

We also found that the occurrence of sarcopenia decreased as levels of physical activities increased. The present findings were similar to prior study showing that PASE score was lower in sarcopenia [24]. A study conducted by Negaresh et al. in older men with sarcopenia vs. nonsarcopenia group undergoing whole-body progressive resistance training program in 8 weeks showed an improvement of quadriceps muscle volume in both groups after training [25]. Meanwhile, in a study conducted by Cunha et al., there was an increased in muscle strength, lean soft tissue, and muscle quality after 12 weeks of resistance training in older women [26]. Prior studies have shown that more physical activity is associated with a delay or prevention of sarcopenia [27].

In this study, IGF-1 levels were lower in the sarcopenia group than the nonsarcopenia group and negatively correlated with sarcopenia status. This is also similar to a study conducted by Bian et al. In their study. they reported that IGF-1 was lower in the sarcopenia group compared with the nonsarcopenia group (all *p* < 0.001) and had a positive correlation with ASM index (*p* < 0.05) [28]. Several studies have found a link between IGF-1 levels and muscle hypertrophy and strength in HD patients [7,29,30]. In an in vivo study, IGF-1 activated a series of anabolic and compensatory pathways, which prevented muscle loss and normal muscle‐nerve interaction, counteracting sarcopenia [31]. IGF-1 regulates protein synthesis and degradation pathways, and changes in IGF-1 signaling in skeletal muscle can dramatically affect myofiber size and function. IGF-1 increases skeletal muscle protein synthesis via PI3K/Akt/mTOR and PI3K/Akt/GSK3*β* pathways. PI3K/Akt can also inhibit FoxOs and suppress transcription of E3 ubiquitin ligases that regulate ubiquitin-proteasome system (UPS) mediated protein degradation [32].

Our results showed elevated myostatin levels in the sarcopenia group than in the nonsarcopenia group. Furthermore, myostatin levels also positively correlated with sarcopenia status. It is currently accepted that myostatin activates several intracellular signaling pathways to inhibit muscle differentiation, decrease protein synthesis, and stimulate protein degradation [33–35]. The upregulation of myostatin in skeletal muscle has been shown as a major pathway responsible for muscle wasting in patients with advanced CKD [33,35]. In CKD patients, an increase in myostatin production can be initiated by complications associated with CKD, such as metabolic acidosis, defective insulin signaling, inflammation, oxidative stress, increased angiotensin II levels, abnormal appetite regulation, and impaired microRNA responses [8,33]. Inflammation in chronic kidney disease or other wasting conditions increases myostatin; meanwhile, increased myostatin can worsen protein-energy wasting conditions and increase its related morbidity [36].

Our study also found that HOMA-IR levels were higher in sarcopenia compared to nonsarcopenia and positively correlated with sarcopenia status. Higher HOMA-IR levels in elderly patients undergoing HD might be explained by diabetes as an underlying disease, aging, or CKD itself. There was decreased sensitivity of myocytes to insulin in aging, which may contribute to diminished muscle mass [37]. In addition, the condition of CKD itself can cause insulin resistance. The etiology of insulin resistance in CKD is multifactorial such as inflammation, oxidative stress, physical inactivity, vitamin D deficiency, metabolic acidosis, adipokine derangement, anemia, and gut microbiome composition [38]. Insulin resistance exists in diabetic and nondiabetic CKD patients, worsening as kidney function declines [39].

A study conducted by Deger et al. showed that skeletal muscle protein synthesis decreased significantly in HD subjects with an insulin resistance state in patients on HD [40]. Insulin and IGF-1 have a similar pathway in muscle metabolism. In muscle, insulin resistance promotes muscle proteolysis and atrophy via regulatory of p85 subunit of Class I phosphatidylinositol 3-Kinase enzyme-protein kinase B (Akt). Insulin resistance also increases the degradation of muscle protein through the UPS pathway. Another factor associated with insulin resistance in CKD is angiotensin II (Ang II), which induces intracellular effects through inflammatory cytokines or reactive oxygen species. As a result, skeletal muscle ATP is depleted, and the ability of AMP-activated protein kinase (AMPK) to replenish energy stores is blocked [41]. A recent study found a novel pathway that IGF-1 or insulin induces regulation of FOXO1 mechanisms, which is important for controlling protein breakdown and preventing muscle atrophy [42].

The main strength of our study is the assessment of sarcopenia according to the most updated definitions and observation of anabolic and catabolic factors that affect protein synthesis and degradation. However, our study has limitations. First, the study population was a small sample size because the study was conducted at a single dialysis center. Our patients may thus not represent general dialysis populations. Second, our study design was cross-sectional, so it is challenging to derive causal relationships from the analysis since the protein synthesis and degradation did not happen simultaneously.

## 5. Conclusions

In conclusion, our results demonstrated that IGF-1, myostatin, and HOMA-IR levels correlated with sarcopenia status in elderly patients undergoing HD. However, further studies of larger populations and longitudinal research are needed to confirm these results.

## Figures and Tables

**Table 1:** Characteristics of patients.

Variable		Total (*n* = 40)	Nonsarcopenia (*n* = 7)	Sarcopenia (*n* = 33)	*p* value		
Age (years)		64 (61–66)	61 (60–66)	64 (61–68)		0.173	
Sex	Male	25(62.5%)	6 (85.7%)	19 (57.6%)		0.224	
	Female	15(37.5%)	1 (2.5%)	14 (42.4%)					
Causes of kidney disease	Diabetes	22(55%)	3 (42.9%)	19 (57.6%)		0.420	
	Hypertension	15(37.5%)	4 (57.1%)	11 (33.3%)					
	Nephrolithiasis	3(7.5%)	0 (0%)	3 (7.5%)					
Duration of HD (months)		48 (24–72)	60 (48–72)	36 (18–72)		0.260	
Blood Pressure (systolic)	≤139 mmHg	18(45%)	3 (42.9%)	15 (45.5%)		0.567	
	140–159 mmHg	18(45%)	4 (57.1%)	14 (42.4%)					
	≥160 mmHg	4 (10%)	0 (0%)	4 (12.1%)					
Laboratory parameters	Hemoglobin (mg/dl)	9.15 (8.32–10.82)	12.4 (11.1–12.5)	8.7 (8.25–9.5)	<0.001^*∗*^		
	BUN (mg/dl)	69.3 ± 22.64	72 ± 23.27	68.72 ± 22.83	0.776		
	Serum creatinine (mg/dl)	11.8 ± 3.74	13.52 ± 3.14	11.43 ± 3.79	0.160		
	Albumin (mg/dl)	3.34 ± 0.26	3.44 ± 0.17	3.32 ± 0.27	0.269		
	Calcium (mg/dl)	8.81 ± 0.69	9.08 ± 0.74	8.75 ± 0.67	0.402		
	Phosphate (mg/dl)	5.7 ± 1.87	6.8 ± 1.61	5.47 ± 1.86	0.064		
	Uric Acid (mg/dl)	7.05 ± 2.07	7.22 ± 1.97	7.01 ± 2.12	0.943		
Sarcopenia parameters	Muscle mass (kg/m^2^)	4.67 ± 1.59	6.63 ± 1.25	4.26 ± 1.34		0.001^*∗*^	
	Muscle mass (men) (kg/m^2^)	5.34 ± 1.46	6.61 ± 1.37	4.94 ± 1.27		0.022^*∗*^	
	Muscle mass (women) (kg/m^2^)	3.57 ± 1.15	N/A	3.34 ± 0.77		0.105	
	Decreased muscle mass	35 (87.5%)	2 (28.6%)	33 (100%)		<0.001^*∗*^	
	HGS	16.45 (10.85–26.85)	27.1 (26.1–29.0)	14.5 (10.0–25.4)		0.005^*∗*^	
	HGS (men) (kg)	26.1 (16.45–27.3)	27.3 (26.7–29.2)	23.5 (14.8–26.9)		0.019^*∗*^	
	HGS (women) (kg)	10 (8.5–12.3)	N/A	10 (8.37–12.07)		0.165	
	Decreased HGS	35 (87.5%)	5 (71.4%)	30 (90.9%)		0.204	
	Gait speed (m/s)	0.81 (0.52–0.83)	0.81 (0.79–1.0)	0.81 (0.45–0.83)		0.148	
	Decreased gait speed	36 (90%)	5 (71.4%)	31 (93.9%)		0.134	
Hormonal parameters	IGF-1 (ng/dl)	37.33 (25.56–50.63)	60.57 (51.31–109.97)	33.43 (23.48–46.01)		0.001^*∗*^	
	Myostatin (ng/dl)	23.74 (18.85–36.26)	18.87 (13.48–20.87)	32.09 (20.52–37.79)		0.010^*∗*^	
	HOMA-IR	2.29 (1.65–2.72)	0.87 (0.56–1.33)	2.38 (1.88–2.79)		<0.001^*∗*^	

Data are shown as %, mean value ± standard deviation, and median (interquartile ranges (Q1-Q3)). *HD*, hemodialysis; BUN, blood urea nitrogen; HGS, handgrip strength; IGF-1, insulin-like growth factor-1; HOMA-IR, homeostasis model assessment-insulin resistance, N/A, not applicable. ^*∗*^Significant at *p* < 0.05.

**Table 2:** Geriatric profiles.

Variable		Total (*n* = 40)	Nonsarcopenia (*n* = 7)	Sarcopenia (*n* = 33)	*p* value
BMI	Underweight (<18.5 kg/m^2^)	0 (0%)	0 (0%)	0 (0%)	0.010^*∗*^
	Normal (18.5–22.9 kg/m^2^)	22 (55.0%)	2 (28.6%)	20 (60.6%)	
	Overweight (23–24.9 kg/m^2^)	11 (27.5%)	1 (14.3%)	10 (30.3%)	
	Obese (>25 kg/m^2^)	7 (17.5%)	4 (57.1%)	3 (9.1%)	

MNA	Adequate nutritional status	8 (20%)	5 (71.4%)	3 (9.1%)	0.002^*∗*^
	At risk of malnutrition	32 (80%)	2 (28.6%)	30 (90.9%)	
	Malnourished	0 (0%)	0(0%)	0(0%)	

MMSE	Normal	31 (77.5%)	1 (14.3%)	8 (24.2%)	1.000
	Mild cognitive impairment	9 (22.5%)	6 (85.7%)	25 (75.8%)	

GDS	Normal	29 (72.5%)	6 (85.7%)	23 (69.7%)	0.650
	Probable depression	11 (27.5%)	1 (14.3%)	10 (30.3%)	
	Depression	0 (0%)	0 (0%)	0 (0%)	

ADL	Independent	26 (65%)	6 (85.7%)	20 (60.6%)	0.420
	Mildly dependent	11 (27.5%)	1 (14.3%)	10 (30.3%)	
	Moderate dependent	3 (7.5%)	0(0%)	3 (9.1%)	

PASE	Sedentary	31 (77.5%)	0 (0%)	31 (93.9%)	<0.001^*∗*^
	Light activity	9 (22.5%)	7 (100%)	2 (6.1%)	
	Moderate to intense activity	0 (0%)	0 (0%)	0 (0%)	

BMI, body mass index; MNA, mini nutritional assessment; MMSE, mini-mental state examination; GDS, geriatric depression scale; ADL, activity of daily living; PASE, physical activity scale examination. ^*∗*^Significant at *p* < 0.05. Comparisons of IGF-1, myostatin, and HOMA-IR in sarcopenia status group.

**Table 3:** Correlation of IGF-1, myostatin, and HOMA-IR with sarcopenia status.

Variable	*r*	*p*
IGF-1	−0.604	<0.001
Myostatin	0.462	0.003
HOMA-IR	0.496	0.001

IGF-1, insulin-like growth factor-1; HOMA-IR, homeostasis model assessment-insulin resistance; *r*, correlation coefficient. ^*∗*^Significant at *p* < 0.05.

## Data Availability

The datasets used and analyzed during the study are available from the corresponding author upon reasonable request.
